# Discontinuation rate of sulfasalazine, leflunomide and methotrexate due to adverse events in a real-life setting (NOR-DMARD)

**DOI:** 10.1093/rap/rkad053

**Published:** 2023-06-16

**Authors:** Pawel Mielnik, Joseph Sexton, Karen M Fagerli, Gunnstein Bakland, Yi Hu, Eirik K Kristianslund, Mari Hoff, Ada Wierød, Tore K Kvien

**Affiliations:** Section for Rheumatology, Department for Neurology, Rheumatology and Physical Medicine, Helse Førde, Førde, Norway; Center for treatment of Rheumatic and Musculoskeletal Diseases (REMEDY), Diakonhjemmet Hospital, Oslo, Norway; Center for treatment of Rheumatic and Musculoskeletal Diseases (REMEDY), Diakonhjemmet Hospital, Oslo, Norway; Department of Rheumatology, University Hospital of Northern Norway, Tromsø, Norway; Lillehammer Hospital for Rheumatic Diseases, Lillehammer, Norway; Center for treatment of Rheumatic and Musculoskeletal Diseases (REMEDY), Diakonhjemmet Hospital, Oslo, Norway; Department of Rheumatology, St. Olavs Hospital, Trondheim, Norway; Department of Neuromedicine and Movement Science, NTNU—Norwegian University of Science and Technology, Trondheim, Norway; Department of Rheumatology, Vestre Viken/Drammen Hospital, Drammen, Norway; Center for treatment of Rheumatic and Musculoskeletal Diseases (REMEDY), Diakonhjemmet Hospital, Oslo, Norway; Faculty of Medicine, University of Oslo, Oslo, Norway

**Keywords:** RA, MTX, LEF, SSZ, discontinuation, adverse events

## Abstract

**Objectives:**

MTX, LEF and SSZ are conventional synthetic DMARDs (csDMARDs) with a well-established role in the treatment of RA. We aimed to estimate and compare the relative risks for adverse events (AEs) and the discontinuation of these drugs owing to AEs.

**Methods:**

We included all 3339 patients from the NOR-DMARD study treated with MTX, LEF or SSZ in monotherapy. All reported AEs were compared between treatment groups using quasi-Poisson regression. In addition, drug retention rates were analysed using Kaplan–Meier estimates with Cox regression to control for possible confounders. We analysed drug retention rates and cumulative risk of discontinuation attributable to AEs using the Kaplan–Meier estimator. We assessed age, sex, baseline DAS in 28 joints with ESR (DAS28-ESR), seropositivity, prednisolone use, previous DMARD use, year of inclusion and co-morbidity as possible cofounders.

**Results:**

We found that the discontinuation rate attributable to AEs was significantly higher for LEF and SSZ than for MTX. After the first year, it was 13.7% (95% CI 12.2, 15.2), 39.6% (95% CI 34.8, 44) and 43.4% (95% CI 38.2, 48.1) for MTX, SSZ and LEF, respectively. Similar results were found when adjusting for confounders. The overall AEs were comparable across the treatment groups. The AE profile was as expected for each drug.

**Conclusion:**

Our work has shown a similar AE profile of csDMARDs to previous data. However, higher discontinuation rates for SSZ and LEF cannot be explained easily from AE profiles.

Key messages

- Treatment discontinuation owing to adverse events is higher for LEF and SSZ than for MTX.
- Non-medical aspects can play a role in patients’ acceptance of adverse events.

## Introduction

MTX, LEF and SSZ are conventional synthetic DMARDs (csDMARDs) with a well-established role in the treatment of RA [1]. SSZ was introduced in the 1940s, the first effective use of MTX was described in the 1960s [2, 3], and LEF was approved for RA in 1998 [4]. Despite this long history and known safety profile, there is a large gap in knowledge about the epidemiology of csDMARD side effects. The authors of the DMARD safety systemic review for EULAR recommendations [5] for the management of RA found only a handful of relevant publications, and owing to heterogeneity, they could not perform meta-analysis on these data.

Our work investigated differences in discontinuation owing to adverse events (AEs) between MTX, LEF and SSZ used in monotherapy, in addition to comparing AE rates between the treatments.

## Methods

We included all 3339 RA patients from the NOR-DMARD study database treated with MTX (*n* = 2379), LEF (*n* = 477) or SSZ (*n* = 483) as monotherapy. The only concomitant treatment allowed was prednisolone. NOR-DMARD is a prospective, observational multicentre study that includes patients treated with DMARDs for inflammatory arthropathies [6]. Patients are included when they start a new treatment and are followed until treatment discontinuation. All participants have to sign informed consent to enter the study. The diagnosis registered in the study is based on clinical judgement. AEs were registered at each study visit by the study nurse or physician and coded according to the Medical Dictionary for Regulatory Activities (MedDRA) coding system [7]. The inclusion period was between 11 December 2000 and 7 June 2012.

We analysed the frequency of AEs overall and according to specific MedDRA categories, such as infections, cardiovascular, respiratory, renal, gastroenterological and dermatological events. The frequencies of severe cardiovascular events, severe infections and malignancies were assessed individually, with severe cardiovascular events defined as any myocardial infarction, unstable angina, cerebral insult including transient ischaemic attack and primary cardiac arrest. Additionally, we evaluated AEs of special interest based on common and well-recognized side effects to evaluate their impact on treatment discontinuation. Selected AEs of special interest included pneumonia, herpes zoster infection, nausea, significant alanine transaminase increase, abdominal pain, diarrhoea, weight loss, oral ulcers, decreased appetite, blood pressure elevation, thrombocytopenia, anaemia, leukopenia, fatigue, headache, depression, muscle pain, any rash and hair loss.

We analysed drug retention rate attributable to AEs using the Kaplan–Meier estimator. In addition, we controlled for potential confounders on the drug retention rate with the Cox proportional hazard model. The following variables were included as confounders: age, sex, baseline DAS in 28 joints with ESR (DAS28-ESR), seropositivity (cumulative positivity for RF and anti-CCP antibodies), concomitant use of prednisolone, previous use of DMARDs, year of inclusion and co-morbidity. The rheumatic disease co-morbidity index (RDCI) [8] was used to express co-morbidities. Owing to missing data for some confounders, 2303 of 3339 patients were included in these analyses. All follow-ups were censored on 31 December 2012.

The relative risk (RR) of AEs was estimated using quasi-Poisson regression with the MTX arm as the reference, controlling for the above set of potential confounders, age, sex, baseline DAS28-ESR, seropositivity, concomitant use of prednisolone, previous use of DMARDs, co-morbidity and duration of observation expressed as a logarithm. All calculations were performed using R v.4.1.1 [9] with the package ‘forestmodel’ [10].

NOR-DMARD has been approved by the regional ethics committee (REK sør-øst, 2011/1339), the Data Inspectorate and the Norwegian Medicines Agency and conducted in accordance with the Declaration of Helsinki.

## Results

Treatment group differences were seen across all examined baseline characteristics (Supplementary Table S1, available at *Rheumatology Advances in Practice* online). There were fewer female patients and more seropositive patients (for RF and/or anti-CCP antibodies) in the LEF group than in the MTX and SSZ groups. LEF patients had higher baseline DAS28-ESR and poorer functional status according to the modified HAQ score. A higher fraction of patients in the MTX and LEF groups used prednisolone, and the average prednisolone dose was higher in those groups than in the SSZ group. The most notable difference was in DMARD naivety. Only 5.5% of LEF patients did not have a prior history of csDMARD use, while the corresponding proportions for MTX and SSZ patients were 62.5 and 49.1%, respectively.

**Figure 1.**
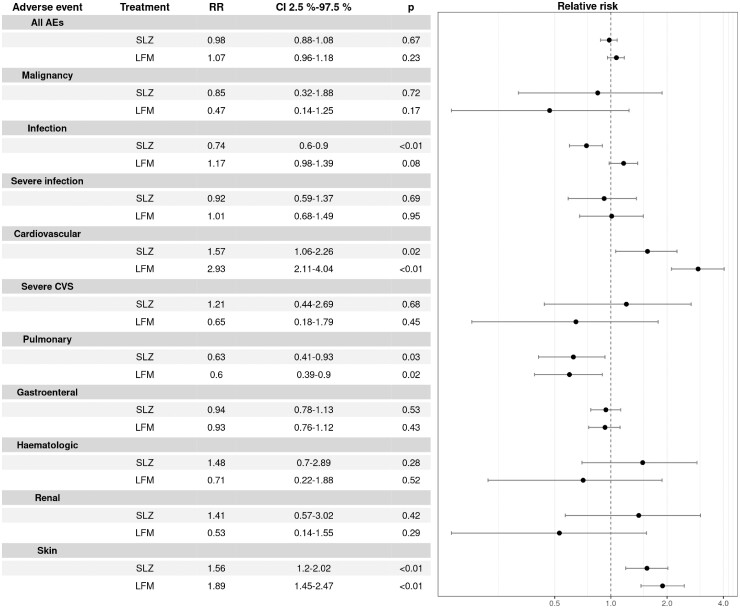
Relative risk for all adverse events and grouped adverse events. The MTX group is a reference, which means that its RR value is 1.0. It is not shown in the diagram. The left panel shows RR values for SSZ and LEF groups, 95% CI 2.5%, 97.5% and *P*-value. The right panel visualizes the RR (filled circles) and CI values (error bars). If an RR with error bars is to the left of the vertical line, the risk of AE occurrence is lower than for MTX; if it is found to the right, the risk is higher. AE: adverse event; RR: relative risk

The overall rate of AEs was similar across groups (RR for SSZ *vs* MTX = 0.98, 95% CI 0.88, 1.08; RR for LEF *vs* MTX = 1.07, 95% CI 0.96, 1.18). Fig. 1 shows the relative risk of the different groups of AEs for SSZ and LEF compared with MTX. SSZ, but not LEF, was associated with a lower infection rate than MTX. A higher rate of overall pulmonary AEs was recorded in the MTX group, whereas skin involvement was less frequent. LEF and SSZ were associated with a higher rate of overall cardiovascular events than MTX. We did not find any difference in the rate of severe infections, severe cardiovascular events or malignancies, nor among overall gastrointestinal, haematological and renal events between the groups (Fig. 1). Serious infections encompassed sepsis, pneumonia, pyelonephritis, meningitis and serious abdominal infection (appendicitis, abdominal abscess). We did not find other infections or cardiovascular events that could be classified as serious in our material.

There were significant differences in the risk of several AEs of special interest between groups (Supplementary Fig. S1, available at *Rheumatology Advances in Practice* online). Not surprisingly, nausea occurred more often in MTX patients, but MTX was less frequently associated with diarrhoea, weight loss and skin rash. The abdominal pain rate in the SSZ group was higher, and the frequency of oral ulcers and hair loss was substantially lower than in the MTX group. There was no difference between MTX and LEF patients for those AEs. LEF, but not SSZ, was associated with a more than nine times higher rate of patients with elevated blood pressure than MTX and a lower rate of patients reporting fatigue. The other AEs of special interest had a similar occurrence rate between drugs.

The discontinuation rate attributable to AEs was significantly higher for LEF and SSZ than for MTX, and after the first year it was 43.4% (95% CI 38.2, 48.1), 39.6% (95% CI 34.8, 44) and 13.7% (95% CI 12.2, 15.2) for each drug respectively. This difference was significant (*P* < 0.0001; Supplementary Fig. S2, available at *Rheumatology Advances in Practice* online).

**Figure 2.**
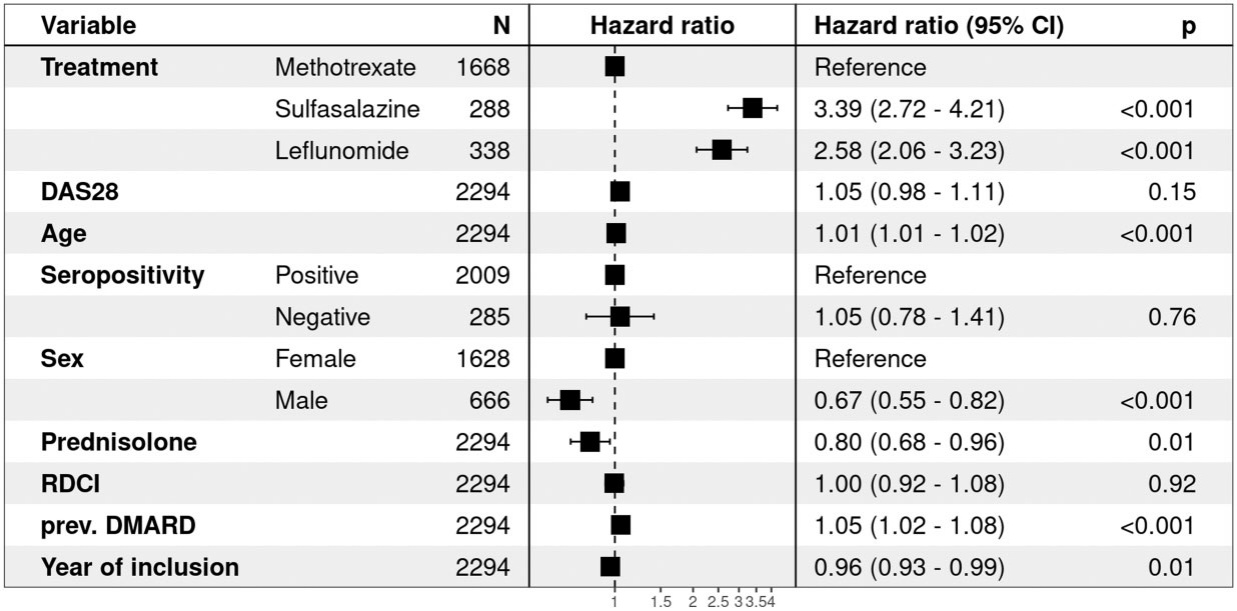
Estimated hazard ratios from Cox proportional hazard model for the treatment groups. The model was adjusted for sex, baseline DAS in 28 joints with ESR (DAS28-ESR), previous DMARD treatment, concomitant prednisolone, seropositivity for anti-CCP antibodies and/or RF, and co-morbidities

The Cox regression analysis confirmed the findings, with a higher risk of discontinuation for SSZ and LEF than for MTX (Fig. 2). Some of the confounders had a significant impact on the treatment discontinuation. Older age and previous use of DMARDs were associated with a higher discontinuation rate, while male gender and concomitant prednisolone use were associated with lower discontinuation rates. There was an association between discontinuation and year of inclusion, with a significantly higher risk in patients included in the first years of the data collection.

## Discussion

We found a higher discontinuation rate attributable to AEs than described in the literature for SSZ [11–13] and LEF [1, 14]. However, the safety profile was similar to previously published data [1, 12, 15]. The differences in AE-related treatment discontinuation cannot be explained easily by observed AE occurrence. Our data did not explain the findings. However, the findings can be interpreted in different ways. Individual patients’ and physicians’ assessments of an AE might be impacted by AE acceptance level, available measures to reduce AEs, and the availability of an alternative treatment. Rheumatologists treating RA can influence patients with milder MTX-associated AEs to continue the treatment more often owing to the higher MTX adoption rate and the fact that it is usually used as concomitant treatment with most biologic DMARDs (bDMARDs). Psychosocial factors can play an essential role in AE acceptance [16], which can also be the case in our study. Patient education regarding treatment benefits, risks and procedures can increase compliance and improve outcomes [17]. In this study, we observed that patients included late in the study, in a period of intensive efforts to develop and implement disease-related patient education, had a lower risk of AE-related treatment discontinuation. Observation spanned the period with the emerging use of bDMARDs. Given that MTX is the first choice in combination with all available bDMARDs, physicians and patients could have more acceptance of MTX with this perspective. The low number of naive patients could bias results for the LEF group despite adjusting to previous treatment. Additionally, the LEF group had a lower functional status and higher disease activity, which could affect outcomes.

No significant difference in the rate of severe AEs was found. The profile of AEs of special interest was as expected. The higher overall risk for cardiovascular AEs in LEF-treated patients can be explained mainly through higher occurrence of hypertension. However, it cannot explain the findings for SSZ. The higher overall pulmonary involvement in MTX patients could be expected but is not explainable by any particular AEs. The higher rate of skin AEs in SSZ and LFM patients is attributable mainly to the higher occurrence of rash; however, we have no more information about the character of the rashes.

The most significant limitation of our study is that we cannot link specific AEs directly with treatment discontinuation. The impact of specific AEs on treatment discontinuation was not registered in the NOR-DMARD study before 2012. It was reported only that discontinuation was ‘due to AE’. We could not use the occurrence date or severity of AE for analysis of discontinuation because it did not necessarily affect the decision discontinue treatment.

The present results supplement data published previously by Lie *et al.* [18]. MTX, LEF and SSZ arms in NOR-DMARD were terminated in 2012, and this manuscript includes a complete NOR-DMARD dataset. We have not published an AE analysis before.

Our work has shown similar profiles of AEs on treatment with MTX, SSZ and LEF to previous research. However, the discontinuation rate attributable to AEs was significantly higher than previously reported, which might reflect local factors, the data collection period or other unknown factors. Further efforts are needed to identify risk factors for csDMARD AEs, in addition to better ways to manage them.

## Supplementary Material

rkad053_Supplementary_Data

## Data Availability

The data underlying this article cannot be shared publicly due to data protection regulations. The data will be shared on reasonable request to the corresponding author.
