# Supplementary material for: Discontinuation rate of sulfasalazine, leflunomide and methotrexate due to adverse events in a real-life setting (NOR-DMARD)
Source: Rheumatol Adv Pract. 2023 Jun 16;7(2):rkad053. doi: 10.1093/rap/rkad053 (PMC10329773; doi:10.1093/rap/rkad053)
Supplement: rkad053_Supplementary_Data [file rkad053_supplementary_data.docx]

Discontinuation rate of sulfasalazine, leflunomide and methotrexate due to adverse events in a real-life setting (NOR-DMARD)

Supplementary materials

Authors:

Pawel Mielnik, Joseph Sexton, Karen M Fagerli, Gunnstein Bakland, Liz P Loli

Eirik K Kristianslund, Erik Rødevand, Ada Wierød , Tore K Kvien

**Supplementary Table S1: Baseline data for all treatment group.**

| Variable | MTX (2379) | SLZ (483) | LEF (477) | **p** |
| --- | --- | --- | --- | --- |
| Gender female (%) | 70.1 | 70.2 | 75.6 | 0.052 |
| Age (years, mean ±SD) | 56.3 (±13.5) | 52.8 (±15.7) | 59.7 (±12.1) | <0.001 |
| Seropositivity (% of patients) | 86.3 | 86.1 | 95.6 | <0.001 |
| Prednisolone use (% of patients) | 67.3 | 52.4 | 63.3 | <0.001 |
| Prednisolone dose (mg, mean ±SD) | 7.2 (±5.1) | 5.9 (±4.7) | 7.2 (±4.8) | 0.001 |
| DMARD naïve patients (%) | 62.5 | 49.1 | 5.6 | <0.001 |
| DAS28-ESR (mean, ±SD) | 4.8 (±1.4) | 5.9 (±1.3) | 7.2 (±1.3) | <0.001 |
| MHAQ (mean, ±SD) | 0.66 (±0.51) | 0.58 (±0.47) | 0.81 (±0.5) | <0.001 |
| CRP (mg/L, mean ±SD) | 21.4 (±26.7) | 16.3 (±24.3) | 24.2 (±25.8) | <0.001 |
| ESR (mm/h, mean ±SD) | 27.9 (±22.0) | 23.9 (±20.0) | 29.8 (±22.8) | <0.001 |

DAS28-ESR – disease activity score with 28 joints and ESR, MHAQ - Modified Health Assessment Questionnaire, MTX – methotrexate, SLZ – slalazopyrine, LEF – leflunomide, DMARD - Disease-modifying antirheumatic drugs, DAS28-ESR disease activity score with 28 joins – erythrocyte sedimentation rate. MHAQ modified health assessment questionnaire CRP – c- reactive protein, ESR – erythrocyte segmentation rte.

Supplementary Figure S1: Relative risk (RR) for AE of special interest.


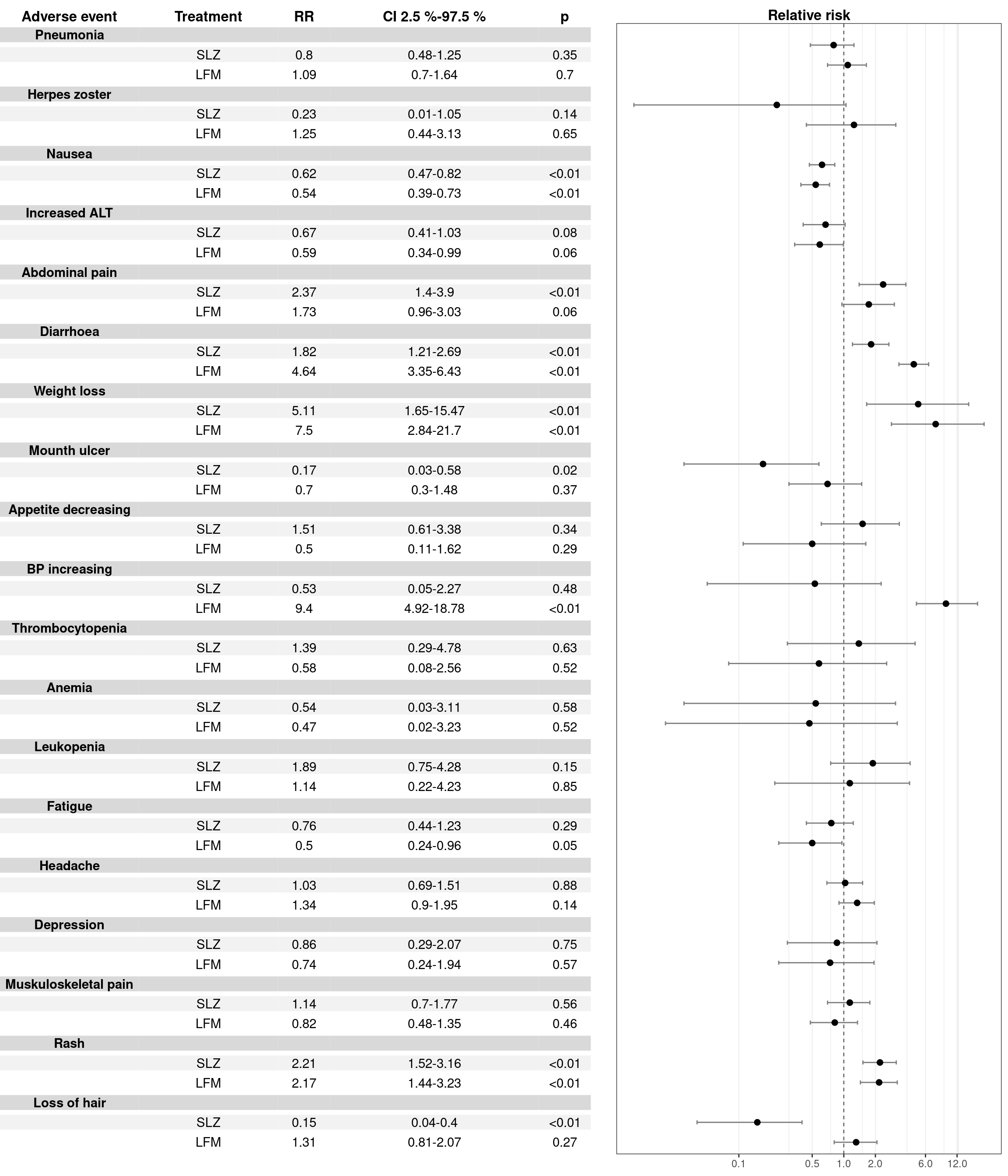


MTX group is the reference, which means RR value is 1.0. It is not shown on the diagram. The left panel shows RR values for SLZ and LEF groups, 95% confidence interval (CI 2.5% - 97.5%) and p-value. The right panel visualises both RR (black point) and CI value (error bars). If RR with error bars finds left to the vertical line, the risk of adverse effect (AE) occurrence is lower than for MTX; if it is found to the right, the risk is higher.

Supplementary Figure S2: Diagram shows cumulative AEs leading to treatment discontinuation from Kaplan-Meier estimation.


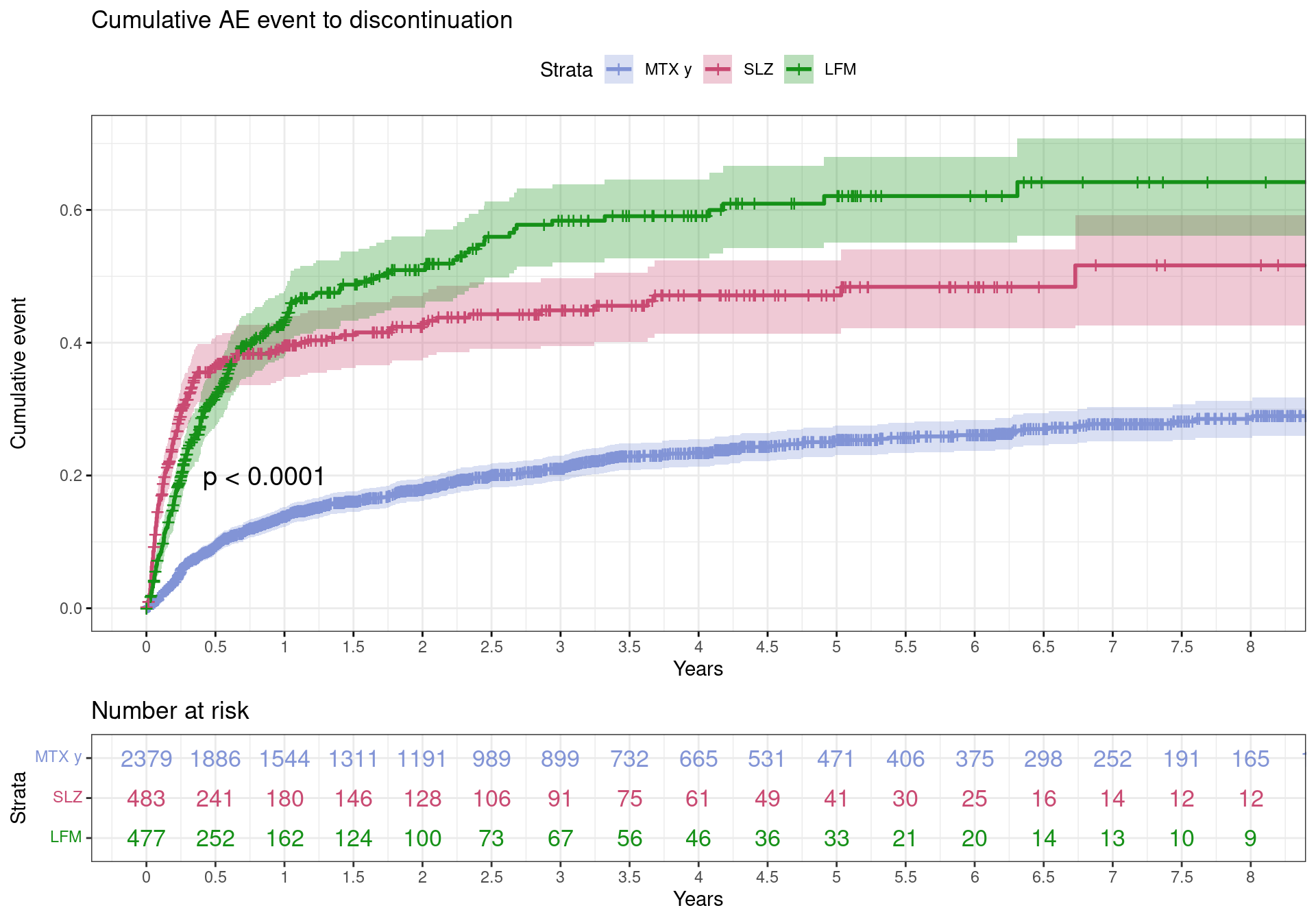


Difference between MTX and to other groups is significant. Shadows show 95% CI. Table under the curve presents the number of patients at risk.
